# Determination of the Mass Fractions of the Heavy Metals in the Recycled Cellulose Pulp

**DOI:** 10.3390/polym16070934

**Published:** 2024-03-29

**Authors:** Mia Klemenčić, Ivana Bolanča Mirković, Nenad Bolf, Marinko Markić

**Affiliations:** 1 Faculty of Graphic Arts, University of Zagreb, 10 000 Zagreb, Croatia; ivana.bolanca.mirkovic@grf.unizg.hr; 2 Faculty of Chemical Engineering and Technology, University of Zagreb, 10 000 Zagreb, Croatiamarinko.markic@fkit.unizg.hr (M.M.)

**Keywords:** recycling paper, pharmaceutical packaging, flotation deinking, heavy metals, IPC-MS

## Abstract

In the process of paper recycling, certain amounts of metals can be found in the cellulose suspension, the source of which is mainly printing inks. The paper industry often uses different technologies to reduce heavy metal emissions. The recycling of laminated packaging contributes to the formation of sticky particles, which affects the concentration of heavy metals. This study aimed to determine the mass fraction of metals in the different phases of the deinking process to optimize the cellulose pulp’s quality and design healthy correct packaging products. In this research, the deinking flotation of laminated and non-laminated samples was carried out by the Ingede 11 method. As a result of the study, the mass fractions of metals in cellulose pulp were divided into four groups according to the mass fraction’s increasing value and the metals’ increasing electronegativity. The quantities of metals were analyzed using Inductively Coupled Mass Spectrometry (ICP-MS). The separation of metals from cellulose pulp is influenced by the presence of adhesives and the electronegativity of the metal. The results of the study show that the recycling process removes certain heavy metals very well, which indicates the good recycling potential of pharmaceutical cardboard samples.

## 1. Introduction

Cellulose is the most abundant natural polymer on Earth [1]. It is a flexible, renewable, and biodegradable raw material, making it widely used in the packaging industry [2,3,4]. All plant matter has, on average, a cellulose concentration of roughly 33% [5]. Cellulose is a complex carbohydrate found in plant cell walls, providing rigidity and strength to plant cells. It is made up of repeating units of β-D-glucose linked together by β-1,4-glycosidic bonds. It has a linear semicrystalline structure containing a long chain of repeated D-glucose units linked by a β-1,4 glycosidic bond between D-glucopyranosyl units. The key process of cellulose pulp production is the extraction of cellulose in its fibrous form [6,7]. Most of the paper today is prepared from the cellulose pulp of coniferous trees (spruce and pines), whose three main components are cellulose, hemicellulose, and lignin [8]. Cellulose has fiber-forming properties due to the presence of straight, long, and parallel chains. It provides strength and stability to the paper structure. Hemicelluloses are a group of polysaccharides that can influence characteristics such as paper porosity, absorbency, and printability. Lignin is a complex polymer that is a natural binding component of wood cells that helps hold cellulose chains together [9]. Paper is a flat material made of plant cellulosic fibers, usually mechanical and/or chemical wood pulp, but also recycled fibers, non-fibrous components (minerals and additives), and water [10]. Different paper products will have different compositions [11,12].

Packaging is known as a protective outside layer, which tends to protect its contents from any physical, chemical, or biological hazards. Food and drugs are subject to the same rules and regulations [13]. Cellulosic paper and paper-based materials are one of the oldest and most widely used food contact materials (FCMs) [14,15]; however, they are porous and offer little resistance to the migration of chemical compounds [16,17]. To improve functional barriers against the transfer of various permeates such as moisture, gases, and lipids through the cellulose wall, they are often treated with additives or laminated [18,19]. Therefore, FCMs are made from raw materials and intentionally added substances (IASs) that extend the service life, but also improve production, stability, mechanical properties, and aesthetics [20,21]. Numerous toxic chemicals such as inks, phthalates, surfactants, bleaches, and hydrocarbons are introduced into paper during the production process [10,22]. Raw materials made from recycled paper and cardboard are more likely to contain some heavy metals as well as other chemical additives [23]. Muncke et al. reported that more than 10,000 chemicals are intentionally used in the production of food contact materials [24].

In paper production, pigments are added to improve the structural and surface properties of the paper. Zinc sulfate is used to increase the opacity of special papers, while zinc oxide is sometimes used to make photocopy paper [25]. Zinc and cadmium pigments are additives that give paper fluorescent properties and increase the cohesive strength of paper surfaces. Zinc is also commonly used in papermaking for fine art applications when white pigments are used [26]. Metals such as copper and aluminum are used for engraving on various packaging [27]. Additionally, the quantity of toxic metals can increase when treating corrugated board packaging using dyes that dissolve in water and an acidic platform without previous surface treatment [25]. The main sources of heavy metals are dyes, which mainly consist of conventional inks and pigments, as well as spot inks and Pantone Matching System (PMS) inks [28]. Inks are considered to increase the content of Cd, Pb, Zn, and Cu [28]. It was concluded that most pigments used in printing inks are based on metal compounds of Zn and Cu, including Pb and Cr, which is why they are already banned for food packaging in some countries. Green and yellow packaging contained compounds such as lead chromate, lead sulfate, and lead oxide [29]. Mohammadpour et al. detected harmful metals such as Pb in high concentrations in most pastry packaging made from recycled paper [30]. In another study, different types of packaging and levels of heavy metals such as Al, As, Ba, Cr, Co, Ni, Pb, and V were analyzed, which in some samples exceeded the permitted concentration [31]. Chang et al. developed a new Liquid Chromatography Mass Spectrometry (LC-MS/MS) analytical method. This method was found to be effective in the rapid analysis of photoinitiators with a high degree of reliability [32]. The ICP-MS method developed for determining the mass fractions of chemical elements (Al, Ba, Fe, Mg, Mn, Pb, Sr, Zn) in paper samples proved to be linear and precise. Values of relative measurement uncertainty ranged from 7.7% to 13.6%, and the used approach allows for improving the quality of data and decision making [33].

However, FCMs may also contain unintentionally added substances (NIASs). The recycled paper contains more total NIAS than virgin paper [34,35]. Identification of these substances is often difficult, sometimes impossible [36,37]. For NIAS screening, substances can be divided into three main groups: volatile, semi-volatile, and non-volatile [38,39]. High-resolution precision mass spectrometry (MS) is a valuable tool for the analysis of non-target substances, including screening and chemical identification, and LC-Orbitrap MS is used to screen suspected migrating compounds in paper food packaging [40,41]. Universal detectors that ensure the detection of the largest possible amount of substances are preferred [39,42]. However, there is no single technique for the assessment of trace metals in materials or their migration, and usually several of them must be combined [16]. In packaging that comes into direct contact with food, contaminants can migrate, that is, chemical compounds from the structure of paper and cardboard packaging can move into the food [28]. This requires a comprehensive analysis of all ingredients that can reach toxicological concentrations in food [16].

In recent years, there have been several research efforts to provide recommendations for the safety of chemicals used in specific packaging and to provide a science-based basis for the development of risk management strategies [40,43,44,45,46,47]. For cardboard and paper packaging products, some standards and regulations limit the content of heavy metals and other harmful substances to ensure safety and environmental protection. In the European Union, the Packaging and Packaging Waste Directive (94/62/EC) provides the framework for limiting the presence of heavy metals in packaging [48]. The directive limits the concentrations of lead, cadmium, mercury, and hexavalent chromium in packaging materials to a maximum value of 100 ppm (mg/kg) for the total content of all four metals combined. This standard applies to all packaging within the EU and to imported packaging material. In the United States, packaging regulations are often state-specific, such as California’s Proposition 65, which requires labeling of products containing chemicals known to cause cancer, birth defects, or other reproductive harm [49]. Although Proposition 65 does not specifically target packaging, its provisions affect packaging materials used for products sold in California. The circular economy is the primary goal of socially responsible and sustainable businesses, but the aforementioned standards emphasize the importance of knowing the composition of recycled raw materials and their suitability for health. 

In this study, the separation of metals from the different stages of deinking flotation, which is carried out using the INGEDE 11 method, from cellulose pulp was investigated. The deinking flotation method is based on the separation of impurities based on their hydrophilicity or hydrophobicity. Cellulose fibers remain in suspension, and hydrophobic impurities and cellulose chains that are shorter in length or are aggregated with adhesives into sticky particles are largely separated in the flotation foam. The study aims to determine the concentration of some heavy metals in a sheet of paper obtained from cellulose pulp and made from cardboard packaging before and after the deinking flotation process. The content of heavy metals in the packaging was analyzed using ICP-MS. The results can be used to determine the quality of the cellulose pulp, i.e., its chemical suitability for use in the production of recycled cellulose. The research results will also show which metals have a lower affinity for separation from the cellulose suspension. If possible, it would be good to omit such metals in the design and production of paper and cardboard products, which can lead to a higher quality of cellulose pulp.

## 2. Materials and Methods

In this study, printing substrate, printed quire, and printed packaging were analyzed. The printing substrate was GC2 cardboard, to which an acrylic polymer-based dispersion was applied. The cellulose and other components of the cardboard printing substrate meet the conditions for food and pharmaceutical products, which means that it does not contain increased concentrations of metals and other harmful components. This is crucial for analyzing the concentration of metals, which is the subject of this research. The printing substrate is also a sustainable product, as it consists of 60% virgin fibers and 30% high-quality recycled fibers. The front side of the printing substrate is coated with a three-layer pigment varnish, while the back side is only coated once.

The laminated printing surface is produced with a dispersion based on acrylic polymers (biaxially oriented polyethylene terephthalate), also known as metalized BoPET film (Ultralen Film GmbH, Rhein, Germany) on the same GC2 cardboard. These self-crosslinking acrylics are free of plasticizers and alkylphenol ethoxylates (APEOs). The APEO compounds can affect the environment, aquatic organisms, and humans. The data show that short-chain compounds have a much lower impact than long-chain compounds [50]. The function of the dispersion is to apply a biaxially oriented polyethylene terephthalate film (BOPET metalized) to obtain a laminated packaging material. The film complies with the requirements of the repealed Directive 20/590/EEC. 

The samples used are laminated and non-laminated substrate, quire, and packaging. The prints were produced on a five-color offset machine with standard UV offset colors from a European manufacturer, white cover offset printing ink, CMYK (cyan, magenta, yellow, black) inks, and dark purple-blue Pantone Matching System (PMS) inks (Zagreb, Croatia). The printing samples were produced using a standard printing form on a Roland 705 five-color offset press machine. The prints were prepared with UV offset inks from recognized European ink factories. The printing form consists of CMYK (cyan, magenta, yellow, and black) and RGB (red, green, and blue) profiles with a raster tonal value of 10 to 100%, full tones of CMYK (cyan, magenta, yellow, and black) and RGB (red, green, and blue colors), and packaging for medicines. The accuracy of the printed form is 83%. The printing process started with white offset printing ink, continued with CMYK separation, and at the end of the printing process, a dark purple-blue Pantone ink was used for the text on the packaging. For rub resistance, the prints were varnished with a UV-curable varnish, a highly reactive photopolymerizable acrylate system that is free of volatile organic compounds (VOCs) and has a low odor and optimal wetting properties. The UV-cured varnish mentioned is VP 1038 high gloss (marked L2). For the assembly of the packaging, an adhesive was applied to the edges that comply with the European Framework Directive 89/109/EEC, the specific regulations for adhesives in the food industry, and the Regulation (EC) of the European Parliament and the Council on materials and articles intended to come into contact with foodstuffs, as well as repealed Directives 80/590/EEC and Commission Regulation (EU) No. 10/2011 on plastic materials and articles intended to come into contact with foodstuffs [51,52].

The samples were disintegrated into cellulose pulp according to ISO 5263-2:2004 [53]. The standard flotation deinking method INGEDE 11 [54] was used to separate the ink particles from the cellulose pulp. The handsheets were produced according to the INGEDE 1 procedure [55] and the ISO 5269-2:2004 standard [56]. The standard handsheets for this study were produced with a device for the automatic production of paper sheets, the Rapid-Köthen-Blattformer sheet former from Frank—PTI, according to the ISO 5269 standard method.

**Figure 1:**
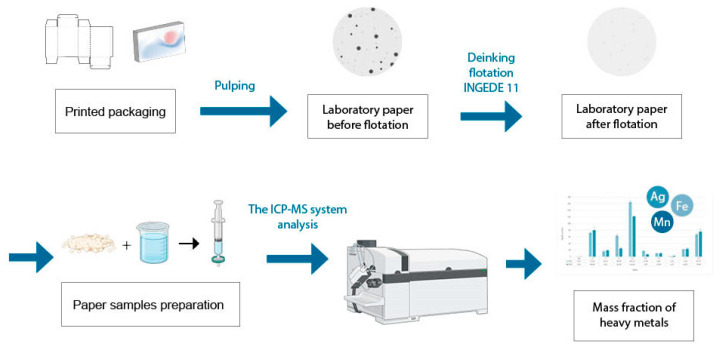
Flowchart of the study.

Figure 1 shows Flowchart of the study, exhibiting all steps of the research. In all procedures, sodium hydroxide was added to the cellulose pulp, giving it an alkaline character (pH = 9.0–11.0). This pH value favors the reactions of saponification and/or hydrolysis of resins from printing inks as well as the swelling of cellulose fibers, which makes them more flexible (better separation of the ink particles). H_2_O_2_ is used to lighten and prevent darkening of the pulp. Therefore, in the procedures with H_2_O_2_ and Na_2_SiO_3_, a silicate is included to prevent the decomposition of H_2_O_2_. The positive properties of Na_2_SiO_3_ are also evident in the reduction in surface tension, the effect on particle dispersion, and the prevention of the binding of impurities.

Table 1 shows samples of laboratory papers made from non-laminated and laminated prints on a cardboard printing base intended for pharmaceutical packaging, before and after the process of separating the ink particles from the cellulose fibers. The quality of the cellulose suspension is also affected by adhesive particles that have formed in the laminated sheet samples and the packaging sample.

### The ICP-MS Analysis

The next step of the study was to determine the metal amounts in the laboratory paper and in the cellulose pulp. The method used for the analysis is inductively coupled plasma mass spectrometry (ICP-MS). The ICP-MS system measures the concentration of elements quantitatively and gives the total amount of each element of interest. The process can be divided into four stages: sample feed, ICP torch, interface, and MS.

The recycled paper sample was cut into small pieces and then weighed to approximately 100 mg. To extract the metals from the paper, 5 mL of hydrochloric acid and nitric acid (1:3 ratio) were added to the sample (J.T. Baker, p.a. purity). After dilution, the analysis was carried out; the sample was filtered with a syringe filter and then diluted ten times. Elemental analysis (Ag, Co, Cr, Cu, Fe, Mn, Ni, Ti, V, Zn analysis) was performed by inductively coupled plasma mass spectrophotometry (ICP-MS PerkinElmer SCIEX ™ ELAN^®^ DRC-e, Concord, ON, Canada), which applies continuous scattering (Table 2). ICP-MS is a tool for analyzing trace metals in environmental samples. In this method, the sample is atomized to generate atomic and small polyatomic ions, which are then detected. The working conditions of the device are listed in Table 2. It is possible to detect metals and various non-metals in liquid samples at very low concentrations.

The ICP-MS calibration was carried out using certified standards. Internal standards are used to compensate for possible measurement deviations. A large number of elements can be detected with an ICP-MS. More than 70 elements can be measured simultaneously in a single analysis. It can measure virtually every naturally occurring element plus many non-natural “radiogenic” isotopes such as technetium, neptunium, plutonium, and americium. The only elements that ICP-MS cannot measure are H and He (which are below the mass range of the mass spectrometer), Ar, N, and O (which are present at high levels from the plasma and air), and F and Ne (which cannot be ionized in an argon plasma) [57]. The advantages of using plasma over other ionization methods, such as flame ionization, are that the ionization takes place in a chemically inert environment, which prevents the formation of oxides, and the ionization is more complete. In addition, the temperature profile of the torch is relatively uniform, which reduces self-absorption effects. Linear calibration curves over several orders of magnitude are observed for ionization processes.

For mass spectrometry, the generation of particles in the submicron range with efficient particle transport to the ICP plays a decisive role [58]. The development and use of the plasma torch as an excitation and ionization source in spectrometry has brought about an important development in analytical elemental analysis. Nowadays, ICP-MS is an essential analytical technique in various fields. This technique requires simple spectra, adequate spectral resolution, and low detection limits for nearly all the elements it can measure. It can detect many elements at levels below 0.1 parts per trillion (ppt). It can also measure elements at concentrations up to 100 s or even 1000 s parts per million (ppm). All metals whose presence was analyzed in this work have a detection limit of up to 10 ppt [57]. For this reason, a mass spectrometer was used as a detector and a high-pressure plasma as an ion source [59]. This method is suitable for the comparison of laboratory sheets, as it is fast, accurate, and precise and allows the analysis of trace elements at low concentrations. The ICP-MS method was used for multi-element analysis for the quantitative determination of silver (Ag), cobalt (Co), copper (Cu), chromium (Cr), nickel (Ni), iron (Fe), manganese (Mn), titanium (Ti), vanadium (V), and zinc (Zn). The mass fraction of all metals is determined according to Formulas (1) and (2).

(1) $$\mathrm{Species}\;\mathrm{mass}\;\mathrm{fraction}\;\mathrm{in}\;\mathrm{the}\;\mathrm{mixture}=Wi=\frac{Mass\;of\;specie\;‘i’\;in\;mixture\;(mg)}{Total\;mass\;of\;mixture\;(kg)}$$

(2) $$\sum_{n=1}^{I=n}Wi=1$$

## 3. Results and Discussion

**Figure 2:**
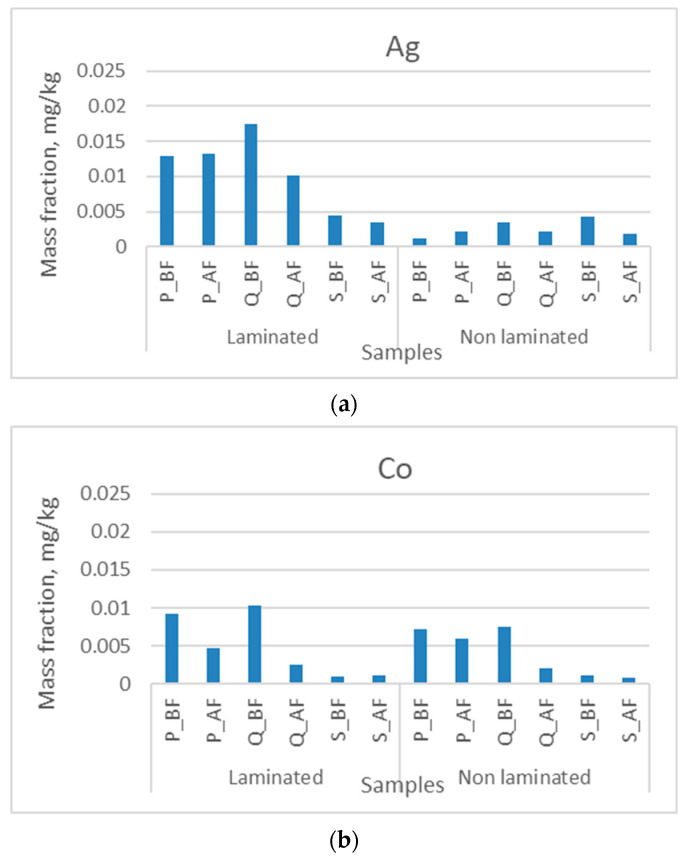
Mass fractions in unlaminated and laminated samples before and after the deinking flotation process for (**a**) silver and (**b**) cobalt.

We have divided the measured mass fractions of metals in laminated and non-laminated samples into four groups so that the graphical representation of the results is as easy to read as possible (mass fractions up to 0.025 mg/kg, 0.04 mg/kg, 0.4 mg/kg, and 7 mg/kg). The mass fractions of silver and cobalt in the unlaminated and laminated samples before and after the deinking flotation process are shown in Figure 2. The mass fractions of silver in the laminated samples are higher than those of the cobalt samples, which is due to the lamination of the sheets. Examination of the mass fractions of the metals in the laboratory paper samples from laminated boxes and sheets before and after deinking flotation leads to the conclusion that cobalt is better separated in the flotation foam. The separation of impurity particles by the deinking flotation method depends on many factors. The quality of the paper collected for recycling, the age of the printed product, and the climatic conditions during its life cycle as well as the coatings, varnishes, laminates, and adhesives can affect their deinkability [12,60,61,62,63,64,65]. Some of these are the small or large size of the ink particles (particles from 10 to 100 μm are best separated), the weak hydrophobicity of the ink particles, the difficulty of separating the particles from the cellulose, the presence of adhesives in the cellulose pulp (formation of sticky particles), and others. The INGEDE 11 method defines the added surfactants sodium hydroxide and sodium silicate, which combine the various necessary functional properties of surfactants and non-surfactants. At the same time, the oleic acid influences the value of the hydrophilic–lipophilic balance (HLB) [66,67]. All this had an impact on the extraction of metal particles from the cellulose pulp, i.e., on the separation from the cellulose fibers. It can be said that the polarity of an atom depends on its electronegativity. In the research, we used the Allred–Rochow electronegativity, which determines the values of the electrostatic force with which the effective nuclear charge acts on the valence electrons. According to the given scale, the electronegativity of the elements is as follows: Ag (1.42), Co (1.70), Cu (1.75), Cr (1.56), Ni (1.75), Fe (1.64), Mn (1.60), Ti (1.32), V (1.45), and Zn (1.66). The value of the effective nuclear charges can be described by the following rule; the higher the charge, the more likely it is to attract electrons. This value is estimated using Slater’s rules.

Z_eff_ = Z − S (3)

Effective nuclear charge (Z_eff_)
Actual nuclear charge (Z)
Shielding constant (S)

Cobalt has a higher electronegativity (1.70) than silver (1.42), which could be associated with better extraction methods for cobalt from cellulose suspensions. Figure 2 shows that the mass fraction of cobalt decreases more than that of silver after the deinking flotation process.

**Figure 3:**
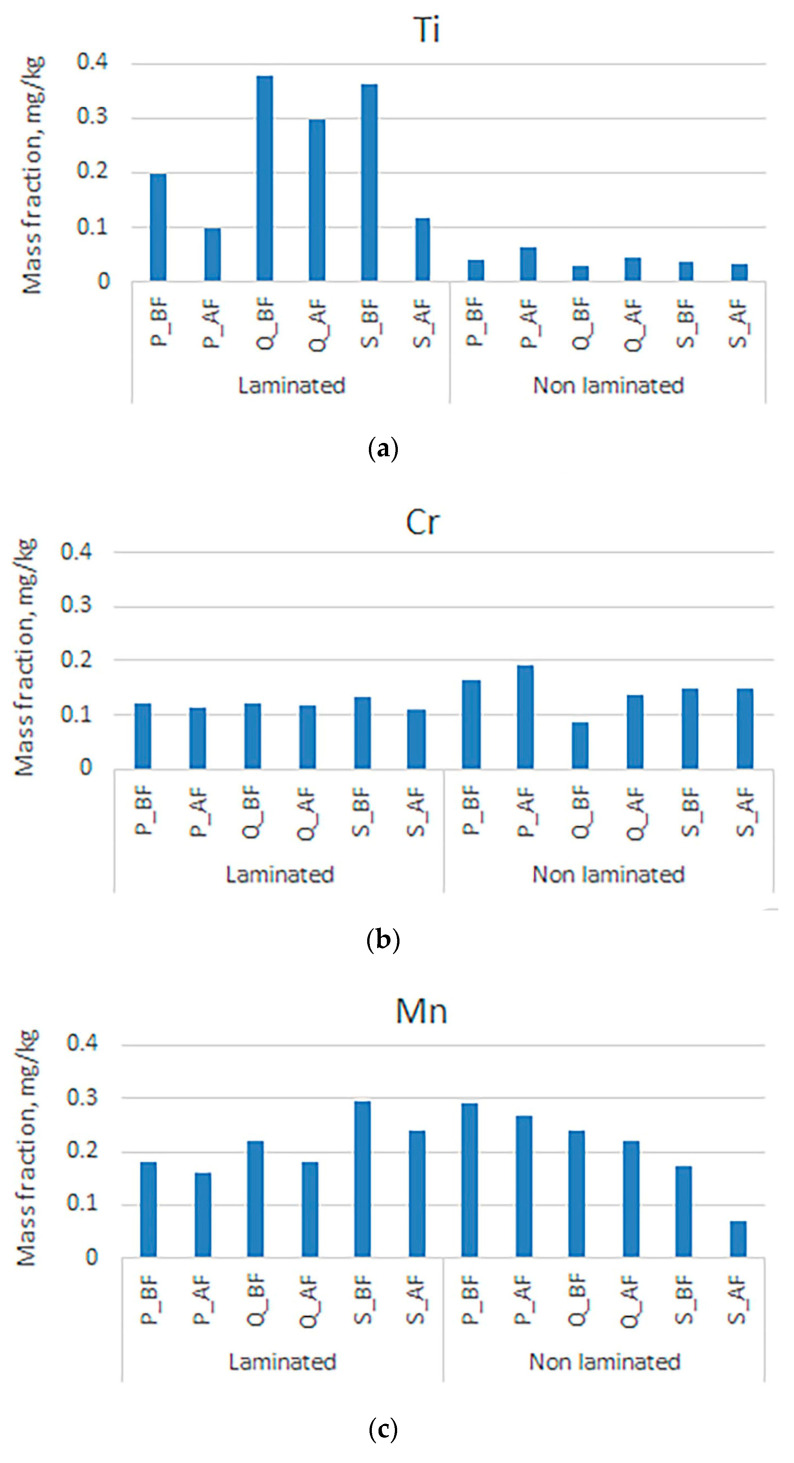

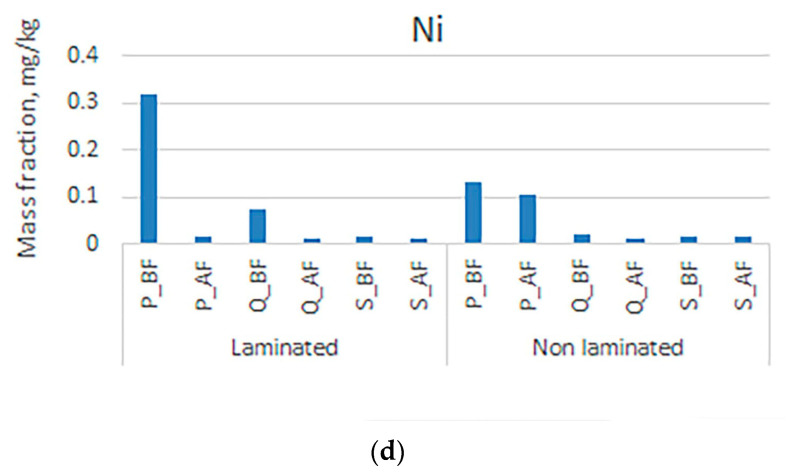
Mass fractions in unlaminated and laminated samples before and after the deinking flotation process for (**a**) titanium, (**b**) chromium, (**c**) manganese, and (**d**) nickel.

When examining the mass fractions of the metals in Figure 3, it is noticeable that the smallest quantities of nickel were detected. It can also be seen that the concentrations in laminated prints are much higher than in non-laminated packaging. It can be assumed that adhesives significantly influence the presence of the mentioned metals, but nickel can be successfully separated by the deinking flotation process. For the samples of manganese and chromium, there is no significant difference between the mass fraction of the metal in laminated and non-laminated samples, but it must be emphasized that slightly higher mass fractions were detected in non-laminated samples. It can be seen that manganese is better separated during deinking flotation, which could also be attributed to the higher electronegativity (1.60). In this series of metals, the least electronegative metal is titanium (1.32), which was found to have significantly higher mass fractions in laminated samples. Increased concentrations of titanium in laminated samples may be associated with the use of white opaque paints applied to the plastic foil for lamination [68,69]. Sticky particles are formed in the cellulose pulp of the laminated samples, to which titanium can adhere and which also contribute to the increase in the mass concentration values. Confirmation of the above can be seen in the low values of the mass fraction of the non-laminated samples.

**Figure 4:**
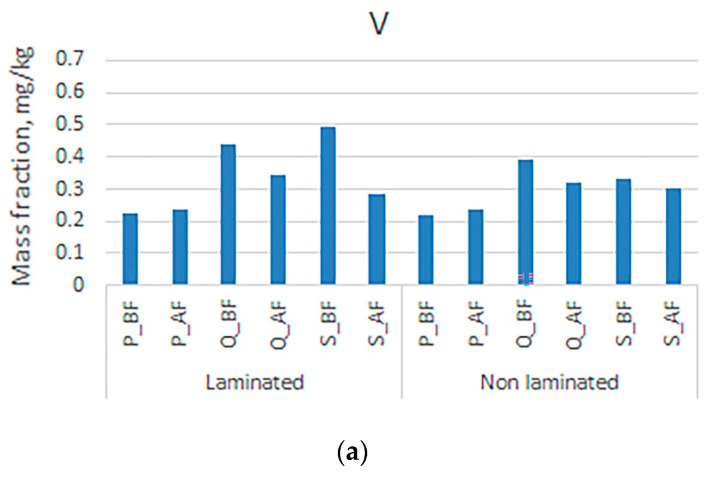

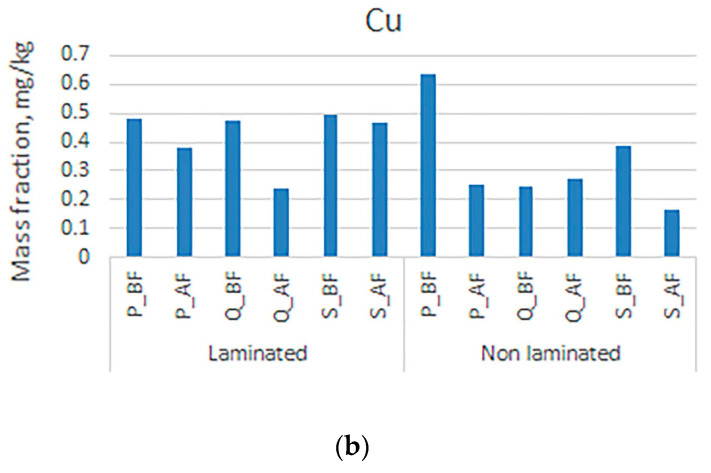
Mass fractions in unlaminated and laminated samples before and after the deinking flotation process for (**a**) vanadium and (**b**) copper.

Figure 4 shows the mass fractions of vanadium and copper. Slightly higher concentrations of copper were detected in samples of laboratory paper sheets. When examining the mass fractions of copper before and after the deinking flotation process, it can be seen that copper separation is most successful in the sample made from non-laminated boxes. From this, it could be concluded that the adhesive for lamination, which is applied to the entire surface of the printing substrate with a laminated box and quire samples, is more effective than the adhesive for gluing the edges of the box. When examining the influence of the type of adhesive mentioned on the extraction of ink particles from cellulose pulp in the deinking flotation process, the trend is the opposite [70]. When examining the mass fractions before and after the deinking flotation process, it can also be seen that the laminating adhesive has no significant influence on the results. It is likely that the lower electronegativity (1.45) and the adhesive attached to the edges of the box contribute to the formation of larger particles, which contribute to a slight increase in the mass fraction of vanadium after the deinking process.

**Figure 5:**
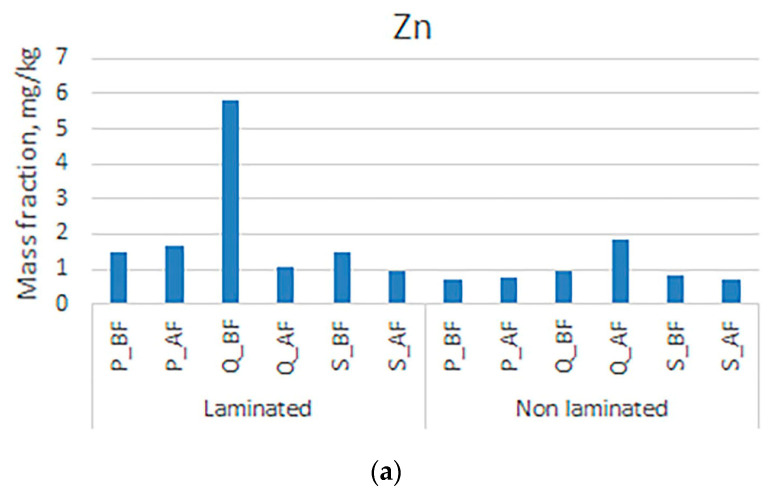

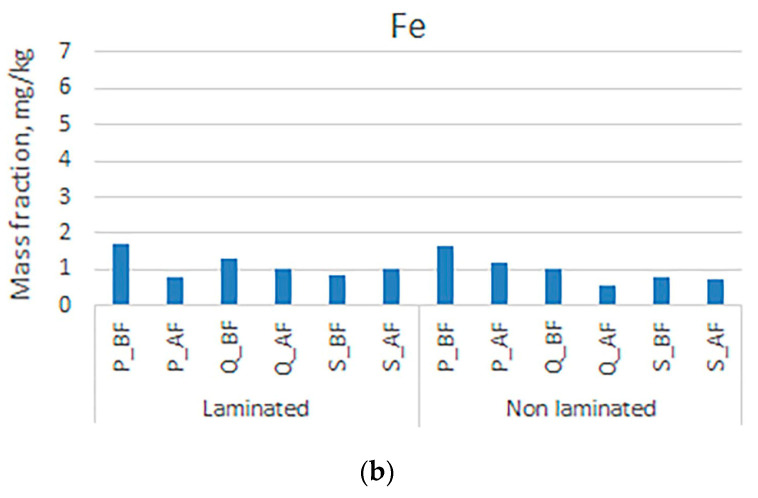
Mass fractions in unlaminated and laminated samples before and after the deinking flotation process for (**a**) zinc and (**b**) iron.

Figure 5 shows that mass fractions of zinc in the samples are slightly higher than those of iron. It can be noticed that the fat content of zinc increases in non-laminated boxes and quires increase after the deinking flotation process. Since no adhesive is present in the mentioned samples, this behavior of zinc could be explained by the lower electronegativity of zinc (1.66), which may negatively affect the extraction process and contribute to its concentration in the cellulose pulp. The literature does not describe frequent occurrences of negative consequences of elevated zinc concentrations for humans, which can lead to gastrointestinal symptoms, reduced absorption of other minerals (copper and iron), immune disorders, neurological symptoms, liver and kidney damage, etc. The diseases mentioned are more likely to be related to occupational exposure than to contamination of food or packaging [71,72]. The results obtained in this research also confirm the facts from the literature about unlikely effects on human health (the mass fraction is below 3.5 mg/kg). The extraction of iron in the process is not among the most efficient in this study, but it has a constant trend of reducing the mass fraction in the cellulose pulp. It should be noted that in the case of iron, the negative influence of the lamination adhesive on the reduction in the mass fraction could be threatened. In support of this, the results confirm the increase in the mass concentration of iron in the samples after the printing substrate deinking flotation process.

From repeated measurements, silver (Ag), cobalt (Co), copper (Cu), chromium (Cr), nickel (Ni), iron (Fe), manganese (Mn), and titanium (Ti) were calculated for all metals. Vanadium (V) and zinc (Zn) values are presented in terms of standard deviation (SD) and variance (σ^2^). Table 3 shows the values for laminated samples, and Table 4 shows the values for non-laminated samples.

Variance is a measure of the dispersion of measured variables, the average sum of the squares of the deviations of the quantity value from the arithmetic mean, while the standard deviation is the positive square root of the variance. Standard deviation is a measure of deviation. It is evident from Table 3 and Table 4 that there are no major deviations obtained in the process of determining the mass fractions of metals, which points to the advantage of the chosen method.

## 4. Conclusions

This research aimed to investigate the composition of pulp and paper concerning the mass fraction of metals, which can help to assess the correctness of the use of recycled cellulose in the production of cardboard packaging in which food or medicines are packaged. By creating databases, the data obtained from the research as well as those that are planned to be obtained in subsequent studies can contribute to the selection of materials that do not contribute to the increase in metals in the cellulose pulp. The recycling of printed materials such as packaging, newspapers, magazines, or other printed products may contain metals in the composition of the paper. The source of the heavy metals is usually in inks and dyes that are applied to the surface of the printing substrate during the printing process. The possible increase in the concentration of certain metals during multiple cycles of recycling would contribute to the health problems of cellulose pulp for the production of packaging products for food and pharmaceutical purposes.

In the study, it has been found that the extraction of metals from cellulose pulp is influenced by the factor of using or not using adhesives and the electronegativity of the metal. We believe that the electronegativity factor is related to the process properties of deinking flotation, which depends on the hydrophilicity and hydrophobicity of the substance. The study shows that the metals Ag, Ti, Cr, V, and Zn, which have a lower electronegativity, have a smaller increase in mass fraction in some phases after deinking flotation. The influence of adhesives and the formation of sticky particles and the influence on the deinking process were also investigated, as were processes related to the separation of ink particles. The results of this study show that the processes for extracting metals from cellulose pulp are significantly influenced by the composition of the adhesive, which should be taken into account in the design of cardboard packaging. Adhesives for lamination have a greater effect on the separation of the mat from the cellulose pulp than the adhesive applied to the edges of the packaging during its assembly. It must be emphasized that the surface on which the lamination adhesive is applied is much larger, so perhaps the reason for this phenomenon is hidden there. In most cases, the deinking flotation method has proven to be a suitable process for the extraction of metals, and the mass fractions of metals measured in the samples do not belong to the categories that would be of concern for human health.

## Figures and Tables

**Table 1:** Samples of laboratory papers made from non-laminated and laminated prints.

Symbol	Sample
P_BF	printed packaging before flotation
P_AF	printed packaging after flotation
Q_BF	printed quire before flotation
Q_AF	printed quire after flotation
S_BF	printing substrate before flotation
S_AF	printing substrate after flotation

**Table 2:** The working conditions of ICP-MS PerkinElmer.

Parameters	Working Conditions
Spray gas flow rate	0.85 L/min
Auxiliary gas flow rate	1.2 L/min
Plasma flow rate	14 L/min;
Lens Voltage	8.5 V; ICP RF
Power supply	1100 W; CeO/Ce = 0.016; Ba^++^/Ba^+^ = 0.015

**Table 3:** Standard deviation and variance of the mass fractions of the metals for the laminated samples.

		Ag	Co	Cu	Cr	Ni	Fe	Mn	Ti	V	Zn
Printed packaging before flotation	SD	0.031	0.125	0.025	0.125	0.025	0.045	0.035	0.025	0.055	0.125
	σ^2^	0.025	0.101	0.001	0.081	0.001	0.031	0.021	0.001	0.041	0.078
Printed packaging after flotation	SD	0.030	0.106	0.006	0.096	0.016	0.036	0.026	0.016	0.032	0.099
	σ^2^	0.021	0.101	0.001	0.071	0.011	0.021	0.021	0.011	0.029	0.077
Printed quire before flotation	SD	0.020	0.085	0.015	0.055	0.025	0.025	0.025	0.025	0.025	0.085
	σ^2^	0.015	0.081	0.001	0.041	0.011	0.011	0.011	0.011	0.016	0.074
Printed quire after flotation	SD	0.020	0.076	0.011	0.056	0.016	0.016	0.016	0.016	0.032	0.075
	σ^2^	0.015	0.061	0.001	0.041	0.011	0.011	0.011	0.011	0.016	0.068
Printed substrate before flotation	SD	0.025	0.055	0.015	0.035	0.025	0.025	0.025	0.025	0.025	0.042
	σ^2^	0.015	0.031	0.001	0.021	0.009	0.011	0.011	0.011	0.011	0.031
Printed substrate after flotation	SD	0.020	0.036	0.006	0.026	0.016	0.016	0.016	0.016	0.022	0.032
	σ^2^	0.011	0.021	0.001	0.015	0.011	0.011	0.011	0.011	0.011	0.016

**Table 4:** Standard deviation and variance of the mass fractions of the metals for the non-laminated samples.

		Ag	Co	Cu	Cr	Ni	Fe	Mn	Ti	V	Zn
Printed packaging before flotation	SD	0.025	0.089	0.025	0.095	0.025	0.035	0.025	0.025	0.045	0.093
	σ^2^	0.020	0.077	0.001	0.075	0.001	0.022	0.011	0.001	0.031	0.067
Printed packaging after flotation	SD	0.025	0.086	0.006	0.066	0.016	0.026	0.016	0.006	0.022	0.073
	σ^2^	0.011	0.071	0.001	0.041	0.011	0.011	0.011	0.011	0.013	0.056
Printed quire before flotation	SD	0.017	0.065	0.015	0.035	0.025	0.020	0.025	0.020	0.020	0.070
	σ^2^	0.011	0.041	0.001	0.021	0.010	0.005	0.001	0.001	0.011	0.055
Printed quire after flotation	SD	0.016	0.056	0.010	0.045	0.015	0.010	0.010	0.010	0.021	0.062
	σ^2^	0.010	0.040	0.001	0.032	0.011	0.001	0.005	0.004	0.015	0.044
Printed substrate before flotation	SD	0.020	0.050	0.015	0.030	0.021	0.015	0.020	0.020	0.015	0.035
	σ^2^	0.011	0.028	0.001	0.015	0.007	0.007	0.001	0.010	0.005	0.025
Printed substrate after flotation	SD	0.020	0.030	0.004	0.020	0.010	0.011	0.010	0.010	0.015	0.025
	σ^2^	0.009	0.017	0.001	0.011	0.007	0.008	0.007	0.008	0.007	0.014

## Data Availability

All data generated or analyzed during this study are included in this manuscript.
